# Antibiotic Stability and Feasibility in Elastomeric Infusion Devices for OPAT: A Review of Current Evidence

**DOI:** 10.3390/jcm14082722

**Published:** 2025-04-15

**Authors:** Chiara Moreal, Luca Martini, Francesca Prataviera, Carlo Tascini, Simone Giuliano

**Affiliations:** 1 Department of Medicine, University of Udine, 33100 Udine, Italy; 2 Clinical of Infectious Disease, Azienda Sanitaria Universitaria Friuli Centrale, 33100 Udine, Italysimone.giuliano@asufc.sanita.fvg.it (S.G.)

**Keywords:** elastomeric pump, antibiotics, OPAT

## Abstract

**Background/Objectives**: Elastomeric infusion pumps have emerged as a transformative tool in outpatient parenteral antimicrobial therapy (OPAT), enabling continuous intravenous administration outside hospital settings, enhancing patient autonomy, reducing healthcare costs, and playing a role in antimicrobial stewardship. This aim of this review is to update current evidence on antibiotic stability in elastomeric infusion pumps, analyzing environmental factors, clinical efficacy, and practical challenges associated with OPAT implementation. **Methods**: A narrative review was conducted using PubMed and the Cochrane Library, focusing on studies published between 2022 and 2025. Included studies assessed antibiotic stability in elastomeric pumps under real-world and laboratory conditions, examining factors such as temperature sensitivity, light exposure, and material interactions. **Results**: Findings indicate considerable variability in antibiotic stability, with some agents maintaining prolonged efficacy while others degrade rapidly under certain conditions. Antibiotics with greater stability are better suited for OPAT, whereas those prone to degradation present challenges for continuous infusion. Clinical studies report favorable treatment outcomes, including high cure rates and manageable adverse event profiles. However, discrepancies between laboratory-controlled conditions and real-world settings highlight the necessity for more comprehensive stability evaluations to ensure optimal antibiotic selection and administration in OPAT programs. **Conclusions**: Optimizing antibiotic formulations, standardizing stability protocols, and advancing elastomeric pump technologies are essential for enhancing OPAT effectiveness. Future research should focus on real-world simulation studies and refining device materials to expand the range of stable antibiotics, ensuring safer and more efficient outpatient antimicrobial therapy.

## 1. Introduction

Elastomeric infusion pumps, commonly referred to as balloon pumps, have emerged as a significant advance in the field of outpatient antimicrobial therapy [1]. These disposable, non-electronic devices consist of a reservoir that, when filled with the prescribed therapeutic agent, exerts a constant pressure in a controlled and continuous manner [2]. This delivery is regulated by a flow restrictor, which ensures precise dosing without the need for external power sources [3]. Such a design makes these devices highly suitable for outpatient settings, particularly within the framework of Outpatient Parenteral Antimicrobial Therapy (OPAT) [4]. The evolution of OPAT has significantly transformed the management of infectious diseases that require prolonged antibiotic administration. Traditionally, patients with infections necessitating intravenous therapy were confined to hospitals for extended periods, leading to increased healthcare costs and a heightened risk of hospital-acquired infections [5].

The introduction of elastomeric infusion pumps has addressed these challenges by enabling continuous drug administration outside the hospital setting, thereby allowing patients to maintain a higher degree of autonomy while reducing overall healthcare expenditures [6]. Moreover, studies have demonstrated that OPAT with elastomeric pumps maintains comparable efficacy to inpatient antibiotic treatment while providing enhanced convenience and patient satisfaction [7]. Additionally, the elastomeric pump offers significant benefits such as reduced hospitalization, cost-effectiveness, and enhanced quality of life [8].

Beyond cost savings and improved patient outcomes, elastomeric pumps play a critical role in antimicrobial stewardship and the fight against multidrug-resistant organisms (MDR). The increasing prevalence of MDR bacteria, such as Pseudomonas aeruginosa and Enterobacteriaceae, presents a significant challenge in hospital settings. OPAT, particularly with elastomeric infusion devices, has been shown to help reduce the selection pressure for resistant pathogens in inpatient environments and limit the transmission of MDR organisms by enabling treatment in outpatient settings [9]. Furthermore, studies highlight the effectiveness of continuous antibiotic infusion using elastomeric pumps in managing infections caused by resistant bacteria, optimizing pharmacokinetics, and ensuring prolonged drug exposure at therapeutic concentrations [10]. Such strategies are particularly relevant for antibiotics requiring extended or continuous infusion, such as β-lactams, which have demonstrated improved efficacy against resistant pathogens when administered via elastomeric devices [9].

Despite these advantages, concerns persist regarding the stability of antibiotics within elastomeric pumps. Stability-related issues can impact both the safety and effectiveness of therapy, as factors such as temperature fluctuations, light exposure, pH variations, and interactions between the medication and the pump material may compromise drug integrity [11].

A comprehensive review by Esteban-Cartelle et al. [1] systematically analyzed the available literature on antibiotic stability in elastomeric infusion devices. The study identified significant variability in antibiotic stability, influenced by factors such as temperature, light exposure, and pH conditions. The review highlighted the poor stability of certain antibiotics, notably amoxicillin, meropenem, and imipenem, while confirming the viability of drugs such as piperacillin/tazobactam and flucloxacillin for prolonged OPAT use. The authors highlighted the need for further high-quality studies that replicate real-life conditions, considering that most stability assessments are conducted under controlled laboratory settings. A further challenge in evaluating antibiotic stability lies in the heterogeneity of study quality and adherence to recognized standards. Regulatory frameworks, including the Yellow Cover Document (YCD), the United States Pharmacopeia (USP), and the British Pharmacopoeia (BP), define specific criteria for determining antimicrobial stability in infusion devices. For instance, the YCD establishes a stricter stability range of 95–105% of the initial drug concentration, whereas many studies apply a broader threshold, considering stability as the retention of at least 90% of the initial concentration. Furthermore, key physical stability parameters, such as pH variations and the formation of visible particulates, are often not consistently assessed, despite their crucial role in ensuring the safety and efficacy of infusion solutions. These concerns align with the findings of Jenkins et al. [3], who systematically reviewed antimicrobial stability in elastomeric devices and highlighted the variability in study methodologies and compliance with NHS YCD standards. Given these considerations, this review aims to consolidate the latest evidence on antibiotic stability in elastomeric infusion devices, focusing on recent advances, validated stability data, newly documented clinical experience and evolving trends in the field. As the need to minimize hospitalizations and curb antimicrobial resistance becomes increasingly urgent, a comprehensive understanding of the evolving role of elastomeric infusion therapy is essential to optimize OPAT strategies and ensure the safe, effective and sustainable use of these devices in clinical practice.

## 2. Materials and Methods

This review follows a narrative review approach, aiming to provide a broad synthesis of available evidence on the stability of antibiotics in elastomeric infusion pumps to facilitate a deeper exploration of key themes and clinical implications. The narrative approach allowed us to integrate the recent findings of two systematic reviews [1,12], with greater contextual depth and practical orientation. A literature search was conducted using PubMed (National Library of Medicine) and the Cochrane Library (Cochrane Database of Systematic Reviews and CENTRAL-Cochrane Central Register of Controlled Trials). The search strategy incorporated keywords including “antibiotic stability”, “elastomeric pump”, “OPAT”, and “portable infusion pumps”. Boolean operators were used to refine the search and ensure the retrieval of studies that specifically examined antibiotic performance in elastomeric devices under both real-world and laboratory conditions. The selection of studies was performed by two independent reviewers, who conducted an initial screening of titles and abstracts, followed by a full-text review of eligible articles. Inclusion criteria required that studies (a) focused on antibiotic stability in elastomeric infusion pumps, (b) evaluated their performance in OPAT settings, (c) were original peer-reviewed full-text articles, and (d) were published between 2022 and January 2025. Studies were excluded if they (a) lacked detailed methodologies or did not specify the type of infusion device used and (b) exclusively examined inpatient settings. To enhance the reliability of findings, data extraction was conducted independently by two reviewers using a standardized data collection framework. Extracted data included information on antibiotic classes, diluent type, stability testing conditions, environmental factors affecting degradation, and reported clinical outcomes. Discrepancies in data interpretation were resolved through discussion, with a third reviewer consulted when necessary.

In the studies included in this review, antibiotic stability was evaluated using different criteria, including the retention of at least 90% of the initial drug concentration over time, variations in pH, the absence of visible particulate formation, and the maintenance of microbiological sterility. Given the heterogeneity in stability assessment methodologies across studies, we have reported findings in accordance with the definitions adopted by each respective study.

## 3. Results

This review included a total of 14 studies examining the stability and clinical outcomes of antibiotics administered via elastomeric infusion pumps in OPAT (Table 1 and Supplementary Table S1).

### 3.1. Stability of Antibiotics in Elastomeric Infusion Pumps

A major focus of the selected studies was the stability and clinical efficacy of antibiotics under different environmental conditions, particularly temperature variations. The studies consistently emphasized the crucial role of temperature in maintaining antibiotic integrity.

#### 3.1.1. Amoxicillin/Clavulanate

The study of Kamalpersad [13] investigates the stability of amoxicillin/clavulanate at various concentrations, pH levels and temperatures. These conditions include those simulating elastomeric infusion in OPAT. At low concentrations (1 mg/mL amoxicillin and 0.2 mg/mL clavulanate), extrapolated shelf-life values at 25 °C were determined to be 22.8 h for amoxicillin and 4.0 h for clavulanate. However, at 40 °C, these values were found to decrease to 4.85 h and 1.38 h, respectively. In refrigerated elastomeric infusers at 2.9 degrees Celsius, a decrease in pH from 8.73 to 6.52 led to a substantial extension in shelf-life, from 72 h to over 263.8 h, for both components. At intermediate concentrations (7.5 mg/mL amoxicillin and 1.5 mg/mL clavulanate), pH adjustment to 7.7 improved amoxicillin stability from 4.2 to 51.8 h and clavulanate from 4.2 to 48.9 h. At clinical concentrations (15 mg/mL amoxicillin and 3 mg/mL clavulanate), even with pH adjustment to 8.4, shelf-life values remained low, with amoxicillin and clavulanate retaining 91.8% and 86.9% of their initial concentrations after 3.8 h and 1.6 h, respectively, at 2.9 °C. Without pH correction, degradation was more rapid across all tested concentrations. Loeuille et al. [11] did not assess amoxicillin/clavulanate nor amoxicillin alone in elastomeric devices. Their evaluation was limited to amoxicillin monotherapy in polyolefin bags at 20 mg/mL in 0.9% NaCl, where it was found to be chemically stable for up to 12 h at room temperature (20–25 °C). However, this setting does not replicate elastomeric infusion conditions, particularly those involving body temperature (37 °C) or polyisoprene materials.

Taken together, current evidence suggests that amoxicillin/clavulanate is highly unstable in elastomeric conditions unless extensively buffered and refrigerated, and its clinical use in OPAT is not advisable without strict pH control and temperature management.

#### 3.1.2. Benzylpenicillin

In the study by Rentsch et al. [13], the stability of benzylpenicillin was evaluated under a range of storage and infusion conditions using elastomeric infusion pumps. It was demonstrated that buffered formulations of benzylpenicillin, prepared with sodium citrate and NaCl 0.9%, demonstrated acceptable stability across all tested concentrations (10, 20, and 40 million IU). Following a period of seven days during which the samples were stored at a temperature of 2–8 °C, the mean concentrations of the buffered benzylpenicillin preparations remained at 97.6% (±1.3%), 96.3% (±0.8%), and 94.9% (±1.1%) of the initial concentration, respectively, for the 10, 20, and 40 million IU concentrations. Subsequent exposure to 37 °C for 24 h resulted in a maximum degradation of 6.5% in the 40 million IU concentration, with all concentrations maintaining ≥94.9% of the initial drug content. In contrast, unbuffered benzylpenicillin diluted in NaCl 0.9% alone exhibited substantial degradation, with mean recovery after seven days at 2–8 °C of only 81%, and undetectable analyte concentrations following an additional 24 h at 37 °C. Stability assessments extending to 48 h at 37 °C revealed progressive degradation in buffered solutions, with measured concentrations decreasing to 86% (±2.0%), 81% (±2.7%), and 63% (±1.6%), respectively, for 10, 20, and 40 million IU formulations.

#### 3.1.3. Cefepime

The stability of cefepime was evaluated at multiple storage temperatures using polyisoprene elastomeric pumps and polypropylene infusion bags. At refrigerated conditions (4 °C), cefepime remained chemically stable for 72 h, maintaining ≥90% of the initial drug concentration. At room temperature (25 °C), the drug remained stable for up to 48 h, with only slight degradation observed after 72 h. At higher temperatures (32 °C), cefepime demonstrated a reduced stability, maintaining ≥90% concentration for 24 h. At 37 °C, cefepime was stable for 24 h but showed significant degradation beyond this period. These findings suggest that while cefepime can be safely used in OPAT, temperature control is critical, and its use should be monitored to avoid prolonged exposure at higher temperatures [14]. These results are consistent with the experimental findings of Loeuille et al. [11], who investigated the stability of cefepime in both polypropylene syringes (at 20–25 °C) and polyisoprene elastomeric devices (at 37 °C). In elastomeric devices at 37 °C (50 mg/mL), cefepime demonstrated rapid degradation, retaining only 83.3% of its initial concentration at 24 h and 59.5% at 48 h, thereby failing to meet chemical stability criteria beyond the first day of infusion. Furthermore, visual modifications were observed after only 6 h at 37 °C, suggesting a potential risk for physical instability under clinical OPAT conditions. Therefore, while cefepime may be suitable for limited-duration infusions at controlled temperatures, its use in elastomeric devices worn close to the body requires strict monitoring and likely limits administration to short-term infusions (≤6–12 h) [11].

#### 3.1.4. Ceftazidime and Ceftazidime/Avibactam

In the study by Fernández-Rubio et al. [14], Ceftazidime (12 g/L) and ceftazidime/avibactam (12/3 g/L) were evaluated for chemical stability in both polypropylene infusion bags and polyisoprene elastomeric pumps under controlled temperature conditions. At 4 °C, both formulations retained over 90% of their initial concentrations for 72 h. At 25 °C, stability was maintained for 48 h, and at 32 °C, for up to 30 h. At 37 °C, the threshold of 90% was exceeded after only 12 h. No significant changes in color or pH were reported during the observation periods. However, despite the acceptable chemical stability, the formation of pyridine, a known degradation product of ceftazidime with potential toxicity, was not quantified. Due to this consideration, the authors recommend limiting in-use durations to 24 h for both ceftazidime and its combination with avibactam, even in settings where stability data might suggest longer infusion times. These findings are partially corroborated by the study of Loeuille et al. [11], who evaluated both ceftazidime and ceftazidime/avibactam under OPAT-like conditions. In polypropylene syringes stored at 25 °C, ceftazidime at 125 mg/mL was chemically stable for up to 24 h in both NaCl and D5W, maintaining ≥90% of the initial concentration. For ceftazidime/avibactam at 125/31.25 mg/mL, similar stability was observed under the same conditions. However, when tested in polyisoprene elastomeric devices at 37 °C, both antibiotics showed significantly reduced stability. Ceftazidime (25 mg/mL) fell below 90% after 8 h in both NaCl and D5W, and ceftazidime/avibactam (25/6.25 mg/mL) dropped below acceptable limits after 12 h in NaCl and even earlier in D5W (unstable beyond 8 h) [11]. Despite the retention of chemical integrity within those shorter durations, Loeuille et al. did not quantify pyridine formation, highlighting a similar safety limitation. Consequently, while ceftazidime and ceftazidime/avibactam may be suitable for use in OPAT with elastomeric devices, infusion durations should be limited to ≤12 h, and preferably to 8 h, particularly at body temperature, pending additional safety evaluations regarding degradation by-products.

#### 3.1.5. Cefiderocol

In the study by Fernández-Rubio et al. [14], cefiderocol was tested at 12 g/L in polyisoprene elastomeric devices filled with 0.9% NaCl and stored at various temperatures. The drug maintained ≥90% of its initial concentration for 72 h at 4 °C, for 72 h at 25 °C, and for 24 h at 32 °C. At 37 °C, the concentration dropped below the 90% threshold after 24 h, indicating that its use in elastomeric devices worn at body temperature should be limited to a maximum of 24 h. In the study by Loeuille et al. [11], the cefiderocol stability was evaluated only in polypropylene syringes at room temperature (20–25 °C), where it was found to be chemically stable for 24 h in both NS and D5W solutions. Therefore, while Fernández-Rubio et al. confirm its short-term suitability for elastomeric infusion at body temperature, the absence of extended data in Loeuille et al. reinforces the recommendation to limit cefiderocol infusion durations to no more than 24 h in OPAT settings.

#### 3.1.6. Ceftolozane/Tazobactam

In the study by Fernández-Rubio et al. [14], the combination Ceftolozane/Tazobactam was tested at 12/6 g/L in polyisoprene elastomeric pumps filled with 0.9% NaCl and stored at different temperatures. The formulation retained ≥90% of its initial concentration for 72 h at 4 °C, 24 h at 25 °C, and 18 h at 32 °C. However, degradation accelerated at higher temperatures, with concentrations falling below the 90% threshold at 37 °C before 24 h, indicating limited suitability for prolonged infusion at body temperature. These findings are corroborated by Loeuille et al. [11], who evaluated ceftolozane/tazobactam at 25/12.5 mg/mL in polyisoprene elastomeric devices. At 37 °C, the combination remained chemically stable (≥90% of initial concentration) for only 12 h. Beyond this point, both components showed significant degradation, with ceftolozane decreasing to 81.4% and tazobactam to 84.3% at 24 h, rendering the solution unsuitable for extended administration under OPAT conditions involving body temperature. Overall, these results demonstrate that ceftolozane/tazobactam is moderately temperature-sensitive, and its use in elastomeric infusion devices should be limited to infusion durations of ≤12 h at 37 °C.

#### 3.1.7. Fosfomycin

The stability of fosfomycin was evaluated under conditions simulating both refrigerated storage and in-use administration at body temperature. At 4 °C, fosfomycin solutions prepared at a concentration of 16 g/250 mL in water for injection remained chemically stable for at least five days in elastomeric pumps, as indicated by degradation remaining within the ±10% threshold defined by ICH guidelines [15]. At 34 °C, two concentrations (16 g/250 mL and 24 g/250 mL) prepared in 5% glucose were assessed. At 16 g/250 mL, the solution retained acceptable stability for six days, with a mean degradation of −2.9% (SD 5.8%) by day five and −12.9% (SD 4.5%) by day nine. At the higher concentration of 24 g/250 mL, degradation was −1.6% (SD 5.3%) at day five and remained within stability limits through day nine (−4.9%, SD 5.5%) [15].

#### 3.1.8. Meropenem and Meropenem/Vaborbactam

Meropenem and its combination with vaborbactam exhibit significant temperature-dependent instability in elastomeric devices, strongly limiting their applicability in OPAT settings. According to Fernández-Rubio et al. [14], both agents were chemically stable for up to 72 h only under refrigerated conditions (4 °C) in polyisoprene elastomeric pumps. At 25 °C, meropenem remained stable for a maximum of 30 h, with degradation increasing thereafter. Meropenem/vaborbactam showed a similar trend, maintaining stability for 30 h at 25 °C, although degradation of meropenem was notably more pronounced than that of vaborbactam. At 32 °C, both meropenem and meropenem/vaborbactam exhibited rapid degradation: meropenem in elastomeric devices fell below the 90% stability threshold within 12 h, with only 71.9% of the initial concentration remaining at 30 h, and as low as 25.6% at 72 h [14]. Meropenem/vaborbactam followed a comparable pattern, with meropenem degrading to 61.5% at 30 h and 25.6% at 72 h, whereas vaborbactam remained more stable (>90%) [14]. At 37 °C, both drugs were considered chemically unstable at all time points. Meropenem degraded rapidly in elastomeric pumps, dropping to 48.9% at 24 h and only 14.5% at 72 h. Vaborbactam retained higher chemical stability, but its combination with meropenem is rendered clinically unviable due to the marked degradation of the carbapenem component [14].

#### 3.1.9. Piperacillin/Tazobactam

Piperacillin/tazobactam is among the most widely used β-lactam/β-lactamase inhibitor combinations in OPAT and demonstrates relatively favorable stability characteristics in elastomeric infusion devices. According to Fernández-Rubio et al. [14], the combination (16 g/2 g per 240 mL) was chemically stable for up to five days at 4 °C, 24 h at 25 °C, and 18–24 h at 32 °C in polyisoprene elastomeric pumps filled with 0.9% NaCl. At 37 °C, degradation became more pronounced, with drug concentrations dropping below the 90% threshold after 12–18 h, thus, limiting use at body temperature unless infusion durations are carefully controlled. Loeuille et al. [11] further evaluated the stability of piperacillin/tazobactam in elastomeric devices under OPAT-like conditions. In their study, the combination was tested at 66.7/8.3 mg/mL in both 0.9% NaCl and D5W, stored at 37 °C in polyisoprene elastomeric pumps. Results showed that the solution retained ≥90% of its initial concentration for up to 12 h in D5W, whereas in NaCl, stability was limited to 8 h. These findings suggest that diluent choice can influence thermal stability, and the use of D5W may modestly extend the safe infusion window at higher temperatures.

#### 3.1.10. Temocillin

Temocillin has demonstrated excellent chemical stability under conditions simulating elastomeric infusion, supporting its use in OPAT. In the study by Fernández-Rubio et al. [16], temocillin at a concentration of 12 g/L, diluted in 0.9% NaCl and stored in polyisoprene elastomeric pumps, maintained ≥90% of its initial concentration for 72 h at 4 °C, 25 °C, and 32 °C, and for 24 h at 37 °C, confirming its suitability for extended infusion durations under a range of temperatures commonly encountered in OPAT settings. The findings are corroborated by Sime et al. [17], who evaluated temocillin at three clinically relevant concentrations (2.17, 16.67, and 25 mg/mL) reconstituted in 0.3% citrate buffer at pH 7, using two different elastomeric devices (Easypump II LT and Dosi-Fusor). Solutions were stored for 14 days under refrigeration (2–8 °C), during which temocillin retained >97% of the initial concentration at all doses and in all devices. During subsequent 24-h exposure at 32 °C, degradation remained minimal, with <9% loss at all concentrations and in both devices [17]. The 95% stability threshold was sustained for at least 12 h in all tested conditions, except at the highest concentration (25 mg/mL) in one device (Dosi-Fusor), where concentration dropped marginally to 94.5% at 12 h. Additionally, Loeuille et al. [11] confirmed that temocillin diluted in 0.9% NaCl and stored in elastomeric pumps at 37 °C retained ≥90% of its initial concentration for 24 h, supporting its feasibility for once-daily continuous infusion in OPAT settings.

### 3.2. Clinical Efficacy of OPAT with Elastomeric Pumps

The clinical effectiveness and safety of OPAT administered via continuous infusion using elastomeric pumps have been evaluated in multiple studies. Outcomes vary by antibiotic agent, patient population, and clinical indication.

#### 3.2.1. Benzylpenicillin (Penicillin G)

Rentsch et al. [13] investigated the feasibility of continuous OPAT with benzylpenicillin (penicillin G), focusing on both in vitro stability and in vivo pharmacokinetics. Their findings highlighted that unbuffered solutions of benzylpenicillin degraded rapidly in elastomeric infusion conditions, whereas citrate-buffered formulations (20 mmol/L, pH 6.5–7.5) preserved chemical integrity for at least 24 h at 37 °C. This supports the feasibility of once-daily administration in elastomeric devices. In vivo pharmacokinetic monitoring in five patients receiving 15–20 million IU/day demonstrated adequate and stable plasma concentrations ranging from 7.2 to 60 mg/L, confirming therapeutic exposure over time without significant fluctuations. Further real-world data are provided by the K-APAT prospective cohort study conducted by Schmidt-Hellerau et al. [18] in Germany, in which 13 of 77 patients (17%) received penicillin G via self-administered continuous infusion using elastomeric pumps. The study included patients with infections such as neurosyphilis, for which benzylpenicillin is the treatment of choice. The median OPAT duration was 15 days, and no catheter-related bloodstream infections were reported. Only one catheter-related complication required medical intervention, and no adverse events were specifically attributed to benzylpenicillin. Clinical outcomes were favorable, with 66% of patients cured and 28% continuing oral sequential therapy at discharge.

#### 3.2.2. Ceftolozane/Tazobactam

The clinical use of ceftolozane/tazobactam administered via continuous infusion through elastomeric pumps in OPAT settings has been evaluated in two complementary studies. In a focused case series, Giuliano et al. [19] reported on seven patients with confirmed Pseudomonas aeruginosa infections treated with continuous infusion of ceftolozane/tazobactam. The majority (71%) of isolates were multidrug-resistant (MDR), including 29% classified as difficult-to-treat resistant (DTR). Infections treated included prosthetic joint infections (43%), osteomyelitis (29%), otomastoiditis (15%), and pneumonia (15%). Ceftolozane/tazobactam was administered in polyisoprene elastomeric pumps at doses of 4.5 g or 9 g daily, diluted in normal saline to a final concentration of 18.75–37.5 mg/mL, and delivered over 24 h. The median treatment duration was 37 days (IQR 25–42) [19]. Clinical cure was achieved in six out of seven patients (86%), with the only treatment failure occurring in a patient with severe comorbidities (Charlson index = 8), for whom OPAT was considered inappropriate. Importantly, no drug-related adverse events were observed. However, two patients (29%) experienced catheter-related complications, including thrombosis and a bloodstream infection, both managed without discontinuation of therapy [19]. These findings were further supported in a broader observational cohort study by Giuliano et al. [20], where 7 of 94 patients (7.5%) treated via elastomeric continuous infusion pumps received ceftolozane/tazobactam. The overall infection cure rate in the full cohort was 88.3%, and adverse events occurred in 12.8% of patients, split equally between drug- and catheter-related causes. Although individual outcomes specific to ceftolozane/tazobactam recipients were not stratified in this larger cohort, the agent was part of a regimen associated with high clinical success and acceptable tolerability. Taken together, these data support the feasibility, efficacy, and safety of ceftolozane/tazobactam in continuous infusion via elastomeric pumps, particularly in patients with MDR P. aeruginosa infections, provided that appropriate source control and patient selection criteria are applied.

#### 3.2.3. Ertapenem

Nosrati et al. [21] examined the use of ertapenem in a retrospective cohort of 98 patients with hidradenitis suppurativa, self-administering the drug over an average of 13.1 weeks. The study found significant clinical improvements, including reductions in HS-PGA scores, pain, and CRP levels. Patient satisfaction was high (80.3%), and 90.8% would recommend the treatment. While no specific adverse events were recorded, the study highlighted concerns regarding potential resistance development with long-term monotherapy.

#### 3.2.4. Flucloxacillin

Flucloxacillin has been evaluated in two key retrospective studies. In the service evaluation by Clarkson et al. [22] involving 39 patients with methicillin-susceptible Staphylococcus aureus (MSSA) infections treated via OPAT, a 74% cure rate was reported, with an additional 21% of patients achieving clinical improvement with complications. Treatment failure occurred in 5% of cases, and adverse events were observed in 20.5%, including acute kidney injury, rash, axillary vein thrombosis, catheter-associated phlebitis, and nausea. Two patients (5%) experienced a relapse within 12 months. In a larger cohort study, Durojaiye et al. [23] retrospectively reviewed 432 OPAT episodes, of which 131 involved flucloxacillin. The overall success rate was 84.3%, and treatment failure occurred in 15.7%. Adverse events were reported in 17.8%, predominantly catheter-related, with drug-related events accounting for 7.6%. Notably, self-administered OPAT was associated with lower failure and readmission rates than nurse-administered therapy. In the prospective K-APAT cohort study from Germany, Schmidt-Hellerau et al. [18] included 77 patients treated via elastomeric pumps, of whom 23% received flucloxacillin. The median OPAT duration was 15 days. Only one patient required intervention for a catheter-related event, and no catheter-related infections were reported. With regard to flucloxacillin, Clarkson et al. [22] conducted a retrospective service evaluation of 39 patients with MSSA infections treated in the OPAT setting. A cure rate of 74% was reported, with an additional 21% of patients achieving clinical resolution with complications. Treatment failure occurred in 5% of cases, while 20.5% experienced adverse events, including acute kidney injury, rash, catheter-associated phlebitis and thrombosis, and nausea. Two patients (5%) experienced a relapse within twelve months. In a larger retrospective cohort, Durojaiye et al. [23] examined 432 OPAT episodes, 131 involving flucloxacillin and 301 with piperacillin/tazobactam. The overall success rate was 84.3%, while treatment failure occurred in 15.7% of cases. Adverse events were reported in 17.8% of episodes, predominantly vascular-access related, with medication-related events accounting for 7.6%. Self- or carer-administered OPAT was associated with significantly lower failure and readmission rates compared to nurse-administered regimens.

#### 3.2.5. Piperacillin/Tazobactam

Piperacillin/tazobactam is one of the most commonly used agents in outpatient parenteral antimicrobial therapy (OPAT) utilizing elastomeric infusion pumps. In a large retrospective study from the UK, Durojaiye et al. [23] evaluated 301 episodes of piperacillin/tazobactam administered via continuous infusion in disposable elastomeric pumps. The median treatment duration was 14 days (IQR 7–34), and the clinical success rate, defined as cure or clinical improvement, was 84.3%. Treatment failure occurred in 15.7% of episodes, although many failures were due to factors unrelated to the antimicrobial regimen, such as unrelated hospital readmissions. Adverse events were reported in 17.9% of episodes, primarily vascular access-related complications, such as line migration and two cases of catheter-associated thrombosis. Only 0.2% experienced line-related infections, and no adverse events were linked to device malfunction. Notably, self- or carer-administered OPAT was associated with a lower treatment failure rate (6.3% vs. 17.4%) and a lower 30-day readmission rate (7.8% vs. 30.4%), underscoring the importance of patient involvement and education. In an Italian observational cohort, Giuliano et al. [20] reported outcomes for 94 patients treated via elastomeric pumps, of whom 29 (30.9%) received piperacillin/tazobactam. The overall infection cure rate was 88.3%, with therapeutic failure in 3.2% and relapse in 2.1%. Adverse events occurred in 12.8% of patients, equally divided between drug-related and line-related complications. The authors highlighted the role of OPAT in personalized care, noting its contribution to cost savings and reduced hospital burden [20]. Further support for piperacillin/tazobactam in OPAT comes from the Spanish study by Rodríguez et al. [24], which reviewed 81 patients treated with elastomeric infusion pumps, of whom 33.3% (n = 27) received piperacillin/tazobactam. The clinical success rate was 85.2%, although the 30-day all-cause mortality rate reached 24.7%. Importantly, multivariate analysis revealed that active cancer within the previous five years (*p* = 0.012) and congestive heart failure (*p* = 0.027) were significantly associated with worse outcomes, while an age of over 80 years old was correlated with better clinical responses (*p* = 0.03). These findings emphasize the importance of comorbidity assessment in selecting OPAT candidates [24]. Together, these studies confirm that piperacillin/tazobactam is a clinically effective and generally safe agent for continuous infusion via elastomeric devices in OPAT, especially when combined with proper patient selection, monitoring, and education.

## 4. Discussion

The integration of elastomeric pumps into OPAT has considerably advanced infection management by enabling the safe administration of continuous intravenous antibiotic therapy outside of the hospital setting. This strategy is aligned with broader healthcare goals, including cost containment, hospital bed optimization, and enhanced patient quality of life. However, its effective implementation is inextricably linked to the physicochemical stability of the antibiotic compounds involved, the reliability of infusion devices, and the clinical outcomes associated with each regimen.

### 4.1. Stability of Antibiotics in Elastomeric Infusion Pumps

A recurrent theme in both historical and recent literature is the challenge posed by real-world variability in OPAT conditions. As highlighted in the systematic review by Esteban-Cartelle et al. [1], significant methodological heterogeneity and frequent non-compliance with NHS YCD standards have undermined the generalizability of laboratory-derived stability data. While many studies apply a threshold of 90% retention of the initial drug concentration to define stability, only a minority (approximately 9%) met YCD’s stricter criteria (95–105%). The more recent evidence reviewed in this article builds upon these findings, affirming the substantial temperature sensitivity of many beta-lactam antibiotics (e.g., cefepime, ceftazidime, meropenem), and reinforcing the critical role of buffer systems, particularly for agents like benzylpenicillin.

Contrary to earlier assumptions in Diamantis et al. [25], which emphasized practical device considerations over compound-specific limitations, newer studies offer more robust, compound-specific pharmacological profiles that better guide clinicians in selecting antibiotics suitable for continuous infusion. For example, the recent demonstration of cefepime’s instability beyond 24 h at 37 °C contrasts with prior, more permissive expectations and underscores the necessity for time-limited administration protocols. Conversely, updated data on buffered benzylpenicillin and temocillin demonstrate a higher level of chemical robustness than previously reported, expanding the therapeutic arsenal for safe OPAT.

Despite these advances, significant discrepancies remain between laboratory and clinical practice. Many studies fail to assess the physical integrity of infusates, such as pH shifts or visible particulates, or to quantify degradation byproducts like pyridine, which are essential for safety evaluations. These omissions may compromise adherence to regulatory expectations, including those of the USP and BP. To enhance real-world applicability, stability assessments must be redesigned to incorporate dynamic environmental simulations (e.g., variable ambient temperatures, patient mobility), aligning laboratory methodologies with OPAT-specific operational realities.

A notable regulatory consideration pertains to the assessment of degradation byproducts, which are crucial for determining the safety of antibiotic formulations over time. The stability of beta-lactam antibiotics varies significantly under different temperature conditions, affecting their suitability for OPAT administration. While stability assessments are often based on the percentage of active pharmaceutical ingredients remaining over time, it is important to note that for certain antibiotics, including ceftazidime, additional regulatory requirements must be considered. Specifically, both the British Pharmacopoeia (BP) and the USP mandate limits on pyridine levels, a degradation byproduct, when determining ceftazidime stability. Studies that do not account for pyridine degradation may not fully comply with these regulatory standards, potentially limiting their applicability in jurisdictions where such requirements are enforced. This underscores the importance of incorporating comprehensive stability assessments that not only evaluate the chemical potency of antibiotics but also monitor the formation of degradation metabolites, ensuring compliance with pharmacopoeial standards and enhancing the clinical relevance of stability studies in OPAT settings.

### 4.2. Clinical Effectiveness and Feasibility Considerations

The reviewed studies collectively affirm the high clinical efficacy of several antibiotics administered via elastomeric infusion, with most agents yielding cure or improvement rates exceeding 80%. This is consistent with earlier findings from Diamantis et al. [25], who reported favorable outcomes with cefoxitin, cloxacillin, and piperacillin/tazobactam. However, more recent evidence allows for greater nuance: specific predictors of clinical failure, such as comorbid malignancy or cardiac dysfunction [24] have now been elucidated, offering clinicians improved patient stratification tools. Among the antibiotics reviewed, temocillin and fosfomycin currently offer the most favorable balance between physicochemical stability and demonstrated clinical efficacy in OPAT, making them strong candidates for once-daily or continuous infusion protocols [16,17,18]. Meropenem and ceftazidime/avibactam highlight a key trade-off in OPAT: while they show strong clinical efficacy—particularly against multidrug-resistant pathogens—their poor thermal stability limits their practical use in continuous infusion without strict temperature control, potentially undermining therapeutic reliability [11,15,21].

Yet, the discussion of treatment failures and adverse events remains underdeveloped in earlier literature. The studies analyzed here emphasize that vascular access complications remain the most common adverse events (12.8% to 17.8%), while drug-related side effects appear less frequent. Importantly, failure is often multifactorial, with device malfunction, inappropriate antibiotic selection, and inadequate patient education acting as co-contributors. This highlights the need for multidisciplinary coordination, structured patient instruction, and frequent reassessment of OPAT suitability, particularly for agents requiring short stability windows or close monitoring.

From a stewardship perspective, the potential impact of OPAT on resistance development remains an important yet underexplored topic. It is often suggested that outpatient treatment may reduce patients’ exposure to hospital-acquired multidrug-resistant organisms, potentially limiting the spread of resistant pathogens in inpatient settings [25] requires further empirical substantiation. Few have systematically monitored resistance emergence, colonization patterns, or microbiome changes over the course of outpatient therapy [19,24]. As a result, while OPAT is presumed to support antimicrobial stewardship goals, its real-world contribution to reducing resistance remains uncertain.

Current data remain largely inferential. Future research could benefit from incorporating microbiological endpoints into prospective OPAT studies, to better characterize the ecological impact of outpatient intravenous therapy and inform stewardship strategies tailored to the unique dynamics of non-hospital environments

### 4.3. Practical Implications and Recommendations for Clinical Practice

The present review identifies a clear need to translate evolving stability and clinical efficacy data into actionable clinical decision-making frameworks. Clinicians selecting antibiotics for OPAT should prioritize agents that demonstrate:

- Proven chemical and physical stability at temperatures approximating 32–37 °C for ≥12 h;
- Low toxicity profiles and minimal degradation byproduct formation;
- Documented clinical success in elastomeric infusion settings;
- Compatibility with available pump materials and volumes.

Buffered benzylpenicillin, temocillin, and piperacillin/tazobactam currently meet many of these criteria. In contrast, antibiotics like meropenem or amoxicillin/clavulanate should be reserved for tightly controlled conditions due to their instability. Where feasible, prefilled refrigerated infusers and shorter infusion durations may extend the usability of otherwise thermolabile compounds. Finally, standardized prescribing protocols that explicitly define drug concentration, diluent, infusion volume, and maximum duration must be integrated into OPAT workflows. Multidisciplinary collaboration, including pharmacists, infectious disease specialists, nurses, and microbiologists, is essential to ensuring that stability, feasibility, and safety converge in patient care.

## 5. Conclusions

The findings of this review underscore the pivotal role of antibiotic stability in ensuring the effectiveness of OPAT with elastomeric pumps. The variability in drug stability across different environmental conditions highlights the necessity of stringent storage protocols and robust patient education. While elastomeric pumps offer a transformative approach to outpatient antimicrobial therapy by enhancing patient autonomy and reducing hospital dependency, the success of this strategy hinges on aligning drug choice with real-world feasibility.

Temperature sensitivity remains a primary concern, necessitating the development of improved insulation methods and controlled storage solutions to mitigate degradation risks, especially for agents with limited thermal stability. Conversely, antibiotics with demonstrated chemical resilience and favorable clinical outcomes, such as temocillin or fosfomycin, represent promising options for routine OPAT regimens.

The high cure rates and manageable adverse events reported across multiple studies support the clinical value of OPAT. However, implementation must account for practical constraints such as infusion duration, device material, and transport conditions, which may significantly impact drug performance and are not always replicated in laboratory settings. Future research should prioritize the standardization of stability testing protocols to facilitate cross-study comparisons and support informed clinical decision-making. Equally important is the need for studies replicating real-world OPAT conditions to bridge the gap between laboratory data and bedside application. Advances in elastomeric device technology, including inert materials and temperature regulation features and smart monitoring, will be critical for broadening the range of viable antibiotics opinions.

Ultimately, integrating these findings into clinical practice requires a pragmatic and evidence-informed approach to antibiotic selection. Matching drug stability profiles with OPAT logistics will not only improve treatment outcomes and patient safety but also strengthen antimicrobial stewardship by reducing reliance on inpatient care and enabling safer use of high-value agents in outpatient settings.

## Figures and Tables

**Table 1:** Stability via an elastomeric infusion pump.

Antibiotic	Diluent	Stable at RT	Stable at 5 °C	Temperature Sensitivity	Clinical Suitability in OPAT
Amoxicillin/Clavulanate [13]	NaCl 0.9% with pH adjustment (HCl 10 M)	<4 h	High if pH adjusted (≥263 h at 2.9 °C)	High	Not suitable without pH adjustment
Benzylpenicillin [14]	NaCl 0.9% (unstable) or Sodium citrate + NaCl 0.9% (stable)	24 h (buffered)	≥7 days (buffered)	High (if unbuffered)	Promising if buffered
Cefepime [11,15]	NaCl 0.9%	48 h	72 h at 4 °C	Rapid degradation at ≥32 °C; <90% at 24 h (37 °C)	Used with caution; short-duration infusions only (≤12 h)
Ceftazidime [11,15]	NaCl 0.9%	48 h	72 h	High: <90% at 12 h (37 °C); <90% at 8 h (37 °C)	Used with caution
Ceftazidime/Avibactam [11,15]	NaCl 0.9%	48 h	72 h	High: <90% at 12 h (37 °C); unstable beyond 12 h (NaCl), 8 h (DW5%)	Used with caution; infusion duration should not exceed 8–12 h
Ceftolozane/Tazobactam [11,15]	NaCl 0.9%	24 h	72 h	Moderate: <90% at 12–18 h at 32 °C1; unstable >12 h at 37 °C	Infusion should be limited to 12 h
Fosfomycin [16]	Water for injection (4 °C) or DW5% (34 °C)	Stable (≥6 days at 34 °C; ≥5 days at 4 °C)	≥5 days	Very Low	Very promising
Meropenem [15]	NaCl 0.9%	3 h	48 h	Very High	Use limited by instability
Piperacillin/Tazobactam [11,15]	NaCl 0.9% or DW5%	24 h	Up to 5 days	Moderate: <90% after 8–12 h at 37 °C in NaCl; better stability in DW5%	Widely used
Temocillin [17,18]	NaCl 0.9%; 0.3% citrate buffer pH 7	24 h	≥14 days	Very low: <5% degradation in 12 h; <10% in 24 h at 32°; stable 24 h at 37°	Excellent candidate for continuous infusion; suitable for once or twice-daily OPAT administration

Legend. DW Dextrose in water; NaCl Sodium chloride; HCl Hydrochloric acid.

## Data Availability

Data collected are available in Supplementary file.

## Supplementary Materials

The following supporting information can be downloaded at: https://www.mdpi.com/article/10.3390/jcm14082722/s1, Table S1: Data extraction.

## Abbreviations

The following abbreviations are used in this manuscript:

OPAT	Outpatient Parenteral Antimicrobial Therapy
MDR	Multidrug-resistant Organisms
SD	Standard Deviation
