# Supplementary material for: Antibiotic Stability and Feasibility in Elastomeric Infusion Devices for OPAT: A Review of Current Evidence
Source: J Clin Med. 2025 Apr 15;14(8):2722. doi: 10.3390/jcm14082722 (PMC12028078; doi:10.3390/jcm14082722)
Supplement: Supplementary file 1 [file jcm-14-02722-s001.zip › jcm-3528747-supplementary.pdf]

Supplementary 1, Table S1. Included studies

**Authors**<br>**Title**<br>**Journal, Year**<br>**Country**	**Study design**<br>**Population**<br>**Therapeutic agent**<br>**Diluent and dosage**	**Stability data**<br>**Clinical efficacy**
Clarkson AM, Snape S.<br><br>Clinical Effectiveness of Continuous Infusion Flucloxacillin in the Outpatient Parenteral Antimicrobial Therapy (OPAT) Setting in a UK Hospital: A Service Evaluation.<br><br>Antibiotics (Basel), 2024<br><br>UK	Retrospective service evaluation<br><br>39 patients with complicated MSSA infections<br><br>Continuous infusion of flucloxacillin<br><br>230 mL in 0.3% w/v citrate-buffered NaCl 0.9%	Not assessed<br><br>Success rate: 74% (n = 29/39); Failure rate: 5% (n = 2/39); Partial success (complicated): 21% (n = 8/39).<br>AEs: Total: 20.5% (n = 8/39); AKI: n = 3, Rash: n = 1; Axillary vein thrombosis: n = 1; Catheter-associated phlebitis: n = 1; Nausea: n = 1; Change of treatment due to new culture (Pseudomonas): n = 1.<br>Relapse within 12 months: n = 2 (5%) 1 due to reintroduction of methotrexate, 1 following source control failure requiring further 12 weeks of IV therapy.<br>Mortality (12-month follow-up): n = 1 (not treatment-related; due to Gram-negative bacteraemia and hepatocellular carcinoma)
Durojaiye OC, Cole J, Kritsotakis EI<br><br>Effectiveness and safety of a disposable elastomeric continuous infusion pump for outpatient parenteral antimicrobial therapy (OPAT) in a UK setting<br><br>J Chemother. 2024<br><br>UK	Retrospective cohort study<br><br>340 patients (432 episodes). Respiratory infections: 38.0% (n = 164/432); Bone and joint infections: 34.5% (n = 149/432); Necrotising otitis externa: 11.6% (n = 50/432); Spinal infections: 8.3% (n = 36/432); Endovascular infections: 3.9% (n = 17/432); Intra-abdominal infections: 1.6% (n = 7/432); Urinary tract infections: 1.4% (n = 6/432); Bacteraemia without focus: 0.7% (n = 3/432)<br><br>Flucloxacillin or piperacillin/tazobactam<br><br>Flucloxacillin: NR<br>Piperacillin/Tazobactam: 0.9% NaCl, 13.5 g/24h	Not assessed<br><br>Success rate: 84.3% (n = 364/432);<br>Failure rate: 15.7% (n = 68/432, unrelated to OPAT)<br>30-day unplanned hospitalisation: 27.1% (n = 117/432, due to non-OPAT-related events)<br>AEs: Total: 17.8% (n = 77/432; 81 events); Vascular access-related: n = 48 (59.3% of AEs; 4.6 events/1000 OPAT-days); Catheter migration: n = 25; Catheter-related thrombosis: n = 2 (0.5%); Line-related infection: n = 1 (0.2%); Medication-related: n = 33 (7.6%; 3.2 events/1000 OPAT-days). Major AEs: n = 29 (6.7%; 2.8 events/1000 OPAT-days). No device-related complications reported. Incomplete infusions: 38.2% (n = 165/432). No significant difference in success rate compared to complete infusions (83.2% vs 86.1%; p = 0.42).
Fernández-Rubio B, Herrera-Hidalgo L, López-Cortés LE, Luque-Márquez R, De Alarcón A, Luque-Pardos S, Fernández-Polo A, Gutiérrez-Urbón JM, Rodríguez-Baño J, Gil-Navarro MV, Gutiérrez-Valencia A<br><br>Stability of temocillin in outpatient parenteral antimicrobial therapy: is it a real option?<br><br>J Antimicrob Chemother. 2023<br><br>Spain	Experimental stability study<br><br>NA<br><br>Temocillin<br><br>6 g daily, diluted in 500 mL 0.9% NaCl, final concentration of 12 g/L, stored in polypropylene infusion bags and polyisoprene elastomeric devices at different temperatures	Stability was assessed using LC-MS/MS following FDA guidelines for drug stability evaluation. Temocillin remained chemically stable for 72 hours at 4°C, 25°C, and 32°C in both polypropylene infusion bags and polyisoprene elastomeric pumps. At 37°C, stability was maintained for 24 hours before dropping below 90%. No precipitation was observed, but minor color changes occurred at higher temperatures.<br><br>Not assessed
Fernández-Rubio B, Herrera-Hidalgo L, de Alarcón A, Luque-Márquez R, López-Cortés LE, Luque S, Gutiérrez-Urbón JM, Fernández-Polo A, Gutiérrez-Valencia A, Gil-Navarro MV<br><br>Stability Studies of Antipseudomonal Beta Lactam Agents for Outpatient Therapy	Experimental stability study<br><br>NA<br><br>Aztreonam, Cefepime, Cefiderocol, Ceftazidime, Ceftazidime/Avibactam, Ceftolozane/Tazobactam, Meropenem, Meropenem/Vaborbactam, Piperacillin/Tazobactam	The study followed FDA guidelines for drug stability assessments. Antibiotics were considered chemically stable if ≥90% of the initial drug concentration was retained.<br>At 4°C: All antibiotics remained stable for 72 hours.<br>At 25°C: All except cefepime, ceftazidime, and ceftazidime/avibactam were stable for 72 hours (these three were stable for 48 hours).<br>At 32°C:<br>• Cefepime stable for 48 hours<br>• Ceftazidime and ceftazidime/avibactam stable for 30 hours

Pharmaceutics. 2023<br><br>Spain	 All antibiotics were diluted in 0.9% NaCl. Aztreonam: 6 g/day (final concentration 12 g/L); Cefepime: 6 g/day (final concentration 12 g/L); Cefiderocol: 6 g/day (final concentration 12 g/L); Ceftazidime: 6 g/day (final concentration 12 g/L); Ceftazidime/Avibactam: 6/1.5 g/day (final concentration 12/3 g/L); Ceftolozane/Tazobactam: 6/3 g/day (final concentration 12/6 g/L); Meropenem: 6 g/day (final concentration 12 g/L); Meropenem/Vaborbactam: 6/6 g/day (final concentration 12/12 g/L); Piperacillin/Tazobactam: 16/2 g/day (final concentration 32/4 g/L)	• Cefiderocol stable for 24 hours<br>• Meropenem and meropenem/vaborbactam stable for 12 hours<br>At 37°C:<br>• Only aztreonam and piperacillin/tazobactam remained stable for 72 hours<br>• Meropenem and meropenem/vaborbactam were unstable at all time points<br><br>Not assessed
Giuliano G, Tarantino D, Tamburrini E, Nurchis MC, Scoppettuolo G, Raffaelli F<br><br>Outpatient parenteral antibiotic therapy (OPAT) through elastomeric continuous infusion pumps in a real-life observational study: Characteristics, safety, and efficacy analysis.<br><br>Enferm Infecc Microbiol Clin (Engl Ed). 2024<br><br>Italy	Retrospective observational cohort study<br><br>94 patients: bone/prosthetic infections: 28.8%, skin/soft tissue infections: 14.9%, bloodstream infections: 14.9%<br><br>Cefepime (34.0%), Piperacillin/Tazobactam (30.9%), Ceftolozane/Tazobactam (7.5%), Oxacillin (7.5%), Meropenem (6.4%), Ceftazidime (3.2%), Cefazolin (3.2%), Colistin (3.2%), Ceftazidime/Avibactam (2.1%), Acyclovir (2.1%)<br><br>Acyclovir: 2.9–3.3 mg/mL (NaCl), Cefazolin: 25 mg/mL (NaCl or DW5%), Cefepime: 25 mg/mL (NaCl or DW5%), Ceftazidime: 25 mg/mL (NaCl or DW5%), Ceftazidime/Avibactam: 25 mg/mL (NaCl), Ceftolozane/Tazobactam: 18.75–37.5 mg/mL (NaCl), Colistin: 37,000 IU/mL (NaCl), Meropenem: 8 mg/mL (NaCl), Oxacillin: 50 mg/mL (NaCl or DW5%), Piperacillin/Tazobactam: 56 mg/mL (NaCl or DW5%)	Not assessed<br><br>Success rate: 88.3% (n = 83/94); Partial success (clinical improvement without confirmed cure): 6.4% (n = 6/94); Therapeutic failure: 3.2% (n = 3/94); Relapse: 2.1% (n = 2/94). Median inpatient treatment duration before OPAT: 14 days (IQR 10–23); Median OPAT treatment duration: 15 days (IQR 8.5–30). Completion of full therapeutic course: 91.4% (n = 86/94). AEs: Total complications: 12.8% (n = 12/94); Drug-related AEs: 6.4% (n = 6); Rash: n = 4 (4.3%); Clostridioides difficile infection: n = 1 (1.1%); Elevated liver function tests: n = 1 (1.1%); Catheter-related AEs: 6.4% (n = 6); Thrombosis: n = 4 (4.3%); Catheter rupture: n = 1 (1.1%); Catheter-related bloodstream infection: n = 1 (1.1%). Hospital readmissions: Total: 10.6% (n = 10/94); Related to infection: n = 5 (5.3%); Related to comorbidities: n = 4 (4.3%); Related to OPAT complication: n = 1 (1.1%)
Giuliano G, Tarantino D, Tamburrini E, Nurchis MC, Scoppettuolo G, Raffaelli F<br><br>Effectiveness and safety of Ceftolozane/Tazobactam administered in continuous infusion through elastomeric pumps in OPAT regimen: a case series<br><br>Infect Dis (Lond). 2024<br><br>Italy	Retrospective case series<br><br>7 patients with Pseudomonas aeruginosa infections (71% of isolates were MDR, 29% were DTR). Prosthetic joint infection: n = 3 (43%); Osteomyelitis: n = 2 (29%); Otomastoiditis: n = 1 (15%); Pneumonia: n = 1 (15%).<br><br>Ceftolozane/Tazobactam<br><br>4.5 g or 9 g per day, diluted in 0.9% NaCl, final concentration: 18.75–37.5 mg/mL	Not assessed<br><br>Success rate: 86% (n = 6/7 patients achieved clinical resolution of infection);<br>Failure rate: 14% (n = 1/7)<br>Median treatment duration: 37 days (IQR 25–42) [71% (n = 5/7) started therapy as inpatients for a median of 19 days (IQR 12–21) before switching to OPAT. 29% (n = 2/7) started OPAT directly following prior inappropriate outpatient antibiotic therapy.]<br>AEs: Total: 29% (n = 2/7); Peri-catheter thrombosis: n = 1; Catheter-related bloodstream infection: n = 1; catheter removed and appropriate therapy initiated. No drug-related AEs reported.
Kamalpersad K, Luna G, Sunderland B, Czarniak P<br><br>An Evaluation of Amoxicillin/Clavulanate Stability in Aqueous Systems, Including Its Suitability for Outpatient Parenteral Antimicrobial Therapy (OPAT)	Experimental stability study<br><br>NA<br><br>Amoxicillin/Clavulanate	Retention of ≥90% of the initial concentration was considered stable, referring to United States Pharmacopeia.<br>Low concentration (1 mg/mL amoxicillin, 0.2 mg/mL clavulanate):<br>• Shelf-life at 40°C: 4.85 hours for amoxicillin, 1.38 hours for clavulanate<br>• Shelf-life at 25°C: 22.8 hours for amoxicillin, 4.0 hours for clavulanate<br>• Lowering pH from 8.73 to 6.52 increased shelf-life at 2.9°C from 72 hours to >263.8 hours<br>Intermediate concentration (7.5 mg/mL amoxicillin, 1.5 mg/mL clavulanate):

Drug Des Devel Ther. 2024<br><br>Australia	Diluition 0.9% NaCl with pH adjustment using 10M hydrochloric acid, from 1 mg/mL to 0.2 mg/mL (pH adjusted from 8.73 to 6.52), to 15 mg/mL / 3 mg/mL (pH adjusted from 8.68 to 8.40)	• Shelf-life at 40°C: 2.74 hours for amoxicillin, 1.03 hours for clavulanate<br>• Lowering pH from 8.85 to 7.69 increased shelf-life at 2.9°C from 4.2 hours to 51.8 hours for amoxicillin and from 4.2 hours to 48.0 hours for clavulanate<br>High concentration (15 mg/mL amoxicillin, 3.0 mg/mL clavulanate):<br>• Shelf-life at 40°C: 0.11 hours for amoxicillin, 0.41 hours for clavulanate<br>• Shelf-life at 25°C: Too short for OPAT feasibility<br>• Shelf-life at 2.9°C: 3.8 hours for amoxicillin, 1.6 hours for clavulanate<br>• Lowering pH further caused precipitation, limiting feasibility for OPAT<br><br>Not assessed
Loeuille G, D'Huart E, Vigneron J, Nisse Y-E, Beiler B, Polo C, Ayari G, Sacrez M, Demoré B, Charmillon A.<br><br>Stability Studies of 16 Antibiotics for Continuous Infusion in Intensive Care Units and for Performing Outpatient Parenteral Antimicrobial Therapy<br><br>Antibiotics, 2022<br><br>France	Experimental physicochemical stability study<br><br>NA<br><br>16 antibiotics including: Amoxicillin, Aztreonam, Cefazolin, Cefepime, Cefiderocol, Cefotaxime, Cefoxitin, Ceftazidime, Ceftazidime/Avibactam, Ceftolozane/Tazobactam, Cloxacillin, Meropenem, Piperacillin, Piperacillin/Tazobactam, Temocillin, Vancomycin<br><br>Syringes: prepared at high concentrations in NaCl, DW5% or SWFI<br>Elastomeric devices: solutions prepared for 120 or 240 mL at 37°C	Defined as ≥90% of initial drug concentration + absence of color/precipitate.<br>In elastomeric devices at 37°C:<br>• 48h: Aztreonam, Vancomycin<br>• 24h: Piperacillin/Tazobactam (DW5%), Temocillin (NaCl)<br>• 8–12h: Ceftolozane/Tazobactam, Ceftazidime/Avibactam (NaCl), Piperacillin/Tazobactam (NS)<br>• <8h or unstable: Meropenem, Cefotaxime, Cefazolin, Cloxacillin, Cefoxitin<br><br>Not assessed
Manca A, Palermiti A, Mula J, Cusato J, Maiese D, Simiele M, De Nicolò A, D'Avolio A<br><br>Stability Study of Fosfomycin in Elastomeric Pumps at 4 °C and 34 °C: Technical Bases for a Continuous Infusion Use for Outpatient Parenteral Antibiotic Therapy<br><br>Pharmaceutics. 2023<br><br>Italy	Experimental stability study<br><br>NA<br><br>Fosfomycin<br><br>Fosfomycin disodium (InfectoFos®). Diluition SWFI and DW 5%	At 4°C (storage temperature before administration):<br>• Fosfomycin remained stable for at least 5 days<br>• No significant degradation was observed within this timeframe<br>At 34°C (external body temperature during administration):<br>• Stable for 6 days at a 16 g/250 mL concentration in elastomeric pumps<br>• At 24 g/250 mL concentration, stability exceeded 9 days (study limit), indicating potential for extended use<br>• On Day 5, degradation was -2.9% (SD 5.8%) for 16 g/250 mL and -1.6% (SD 5.3%) for 24 g/250 mL<br>• On Day 9, degradation was -12.9% (SD 4.5%) for 16 g/250 mL and -4.9% (SD 5.5%) for 24 g/250 mL<br><br>Not assessed
Nosrati A, Ch'en PY, Torpey ME, Shokrian N, Ball G, Benesh G, Andriano TM, Zhu TR, Heibel HD, Hosgood HD, Campton KL, Cohen SR<br><br>Title: Efficacy and Durability of Intravenous Ertapenem Therapy for Recalcitrant Hidradenitis Suppurativa<br><br>JAMA Dermatology, 2024<br><br>USA	Retrospective cohort study<br><br>98 with recalcitrant hidradenitis suppurativa<br><br>Ertapenem<br><br>1 g IV daily via a 100-mL elastomeric pump (Avanos Medical, Inc.), self-administered at home through a PICC for 12–16 weeks. Diluent NR	Not assessed<br><br>Significant clinical improvements from baseline to posttherapy follow-up: hidradenitis Suppurativa Physician Global Assessment score: Improved from 3.9 (SD 1.0) to 2.7 (SD 1.2) (P < .001); numerical rating scale for pain (0–10 scale): Decreased from 4.2 (SD 3.3) to 1.8 (SD 2.7) (P < .001); C-reactive protein: from 5.4 mg/dL (SD 11.4) to 2.4 mg/dL (SD 2.0) (P < .001); Interleukin-6 (IL-6): from 25.2 pg/mL (SD 21.1) to 13.7 pg/mL (SD 13.9) (P < .001); leukocyte count: from 11.34 (SD 3.9) to 10.0 (SD 3.4) (P < .001).<br>Patient Satisfaction: 78% of patients (76/98) participated in a follow-up survey. 80.3% reported medium-to-high satisfaction with treatment. 90.8% would recommend ertapenem therapy to others.<br>AEs: 9.2% PICC line complications requiring replacement; 7.1% developed dermatitis at the PICC insertion site; 8.2% experienced diarrhea, 5.1% nausea, 2% headaches, 1% candidiasis, and 1% syncope.
Rentsch KM, Khanna N, Halbeisen D, Osthoff M	Experimental in vitro stability study + in vivo TDM	Unbuffered benzylpenicillin degraded significantly even at refrigerated temperatures (2–8°C).<br>After 7 days at 4°C, only 81% of the original concentration remained.<br>After 8 days, the drug was completely degraded and undetectable.

Enhancing Stability and Investigating Target Attainment of Benzylpenicillin in Outpatient Parenteral Antimicrobial Therapy: Insights from In Vitro and In Vivo Evaluations<br><br>Antibiotics (Basel). 2024<br><br>Switzerland	5 patients for conditions such as infective endocarditis<br><br>Benzylpenicillin<br><br>Benzylpenicillin administered at 10 MIU, 20 MIU, or 40 MIU concentrations. Diluents NaCl 0.9% or sodium citrate solution plus NaCl 0.9%	Buffered benzylpenicillin (sodium citrate) was much more stable:<br>At 4°C for 7 days, degradation was ≤2.2% across all concentrations.<br>At 37°C for 24 hours, stability was ≥94.9% for all concentrations:<br>• 10 MIU: 97.6% (±1.3%) remaining<br>• 20 MIU: 96.3% (±0.8%) remaining<br>• 40 MIU: 94.9% (±1.1%) remaining<br>At 37°C for 48 hours, degradation increased:<br>• 10 MiIU: 86% remaining<br>• 20 MIU: 81% remaining<br>• 40 MIU: 63% remaining<br>Degradation products included penicilloic acid, penillic acid, and penilloic acid, known to be allergenic and toxic at high concentrations.<br><br>TDM in 5 patients: Continuous infusion resulted in sufficient-to-high plasma levels of benzylpenicillin; levels in intermittent bolus dosing were suboptimal (0.5–6.6 mg/L); during OPAT, levels increased to 7.2–60 mg/L, ensuring sustained efficacy; no significant inter-individual variability was found ($p = 0.85$); benzylpenicillin was well-tolerated, but one patient with renal impairment showed an 18% increase in creatinine levels
Rodríguez SF, Legaspi YC, Romay LEM, Ayuso García B, Castellano Copa P, Peinó Camba P, Barcia Losada A, Rodríguez Díaz C<br><br>Retrospective Study of Home Antibiotic Infusion Therapy in Elastomeric Infusion Pumps.<br><br>Farmacia Hospitalaria, 2024<br><br>Spain	Retrospective observational study<br><br>81 patients: respiratory infections (27.2%), bacteremia (16%), skin and soft tissue infections (12.3%), urinary tract infections (11.1%), endocarditis (9.9%), osteoarticular infections (7.4%), intra-abdominal infections (3.7%), CNS infections (1.2%)<br><br>Ampicillin (7.4%), Cefazolin (16%), Cefepime (3.7%), Ceftaroline (1.2%), Ceftazidime (24.7%), Ceftazidime/Avibactam (3.7%), Meropenem (10%), Piperacillin/Tazobactam (33.3%).<br><br>Diluition NR	Not assessed<br><br>Success rate: 85.2% (n=69/81)<br>Microbiological findings: Pseudomonas aeruginosa was the most frequently isolated pathogen (39.6%), Enterobacteriaceae were present in 23.6% of cases (including E. coli, K. pneumoniae, Morganella morganii), MRSA was rare (n=1), while MSSA was isolated in 11 patients. Therapeutic failure: 22.2% within 30 days<br>AEs: 16% of patients experienced venous access complications (extravasation, loss of IV line, mild phlebitis), nephrotoxicity (n=1) sage adjustment.
Schmidt-Hellerau K, Baade N, Günther M, Scholten N, Lindemann CH, Leisse C, Oberröhrmann C, Peter S, Jung N, Suarez I, Horn C, Ihle P, Küpper-Nybelen J, Hagemeier A, Hellmich M, Lehmann C.<br><br>Outpatient parenteral antimicrobial therapy (OPAT) in Germany: insights and clinical outcomes from the K-APAT cohort study.<br><br>Infection. 2024<br><br>Germany	Prospective observational cohort study<br><br>77 patients (Joint and bone infections (26%), Staphylococcus aureus bloodstream infections (21%), Infectious endocarditis (14%), Neurosyphilis (12%), Pulmonary infections (4%), Other severe infections (32%).<br><br>Caspofungin (4%), Cefazolin (5%), Ceftriaxone (13%), Flucloxacillin (23%), Fosfomycin (5%), Meropenem (8%), Penicillin G (17%), Vancomycin (10%), Others (22%).<br><br>Diluition NR	Not assessed<br><br>Cured at end of OPAT: 66% (49/74 patients); Switched to oral sequential therapy: 28%, Treatment failure: 0%, Mortality during OPAT: 4% (3 patients), Lost to follow-up: 4% (3 patients). AEs: Severe catheter-related complications in 5% of patients (mostly related to PICC line displacements), No diagnosed catheter-related infections, Rehospitalization rate: 21% before completion of OPAT, with 56% of these cases related to the underlying disease.
Sime FB, Wallis S, Jamieson C, Hills T, Gilchrist M, Santillo M, Seaton RA, Drummond F, Roberts J<br><br>Evaluation of the Stability of Temocillin in Elastomeric Infusion Devices Used for Outpatient Parenteral Antimicrobial Therapy in Accordance	Experimental stability study<br><br>NA<br><br>Temocillin	At 5°C (Refrigerated Storage):<br>• Stable for 14 days in both elastomeric devices across all concentrations.<br>• Percentage of temocillin remaining exceeded 97% in all conditions.<br>At 32°C (In-Use Temperature for 24 h):<br>• Maintained >95% stability for at least 12 hours at all concentrations.<br>• High concentration (25 mg/mL) in the Dosi-Fusor device only met this criterion for 10 hours.

with the Requirements of the UK NHS Yellow Cover Document<br><br>European Journal of Hospital Pharmacy, 2023<br><br>United Kingdom	Reconstitution with 30 mM sodium phosphate buffer solution at pH 7	• After 24 hours, degradation remained <9% for all devices and concentrations.<br>Degradation Products:<br>• Five degradation peaks were identified, corresponding to penicilloic acid and penillic acid derivatives.<br>• No significant subvisible particle formation was detected.<br>pH Stability:<br>• Maintained pH ~7.0 in refrigerated storage.<br>• Slight pH drop during 32°C exposure, with a final range of 6.26–6.51 after 24 hours.<br><br>Not assessed

Legend. AKI Acute kidney injury; CNS central nervous system; DTR Difficult to treat-resistant; DW dextrose in water; FDA Food and Drug Administration; h Hours; IV intravenous; LC-MS/MS: Liquid Chromatography-Tandem Mass Spectrometry; MDR multidrug resistant; MRSA Methicillin-Resistant Staphylococcus aureus; MSSA Methicillin-Sensitive Staphylococcus aureus; NA Not applicable; NaCl sodium chloride; NR Not reported; OPAT Outpatient Parenteral Antimicrobial Therapy; PICC Peripherally Inserted Central Catheter; RT Room Temperature; SD standard deviation; SWFI Sterile Water for Injection; TDM therapeutic drug monitoring; YCD Yellow Cover Document.
